# Opportunities for improving use of evidence‐based therapy in patients with type 2 diabetes and cardiovascular disease

**DOI:** 10.1002/clc.23252

**Published:** 2019-08-26

**Authors:** Yumin Gao, Eric Peterson, Neha Pagidipati

**Affiliations:** ^1^ Department of Epidemiology Johns Hopkins Bloomberg School of Public Health Baltimore Maryland; ^2^ Department of Medicine, Duke Clinical Research Institute, Center for Preventive Medicine Duke University Durham North Carolina

**Keywords:** barriers to care, cardiovascular disease, implementation science, type 2 diabetes

## Abstract

Evidence‐based therapy that target hyperlipidemia, hypertension, smoking cessation, and weight loss have demonstrated significant benefits in reducing cardiovascular risks and related events. Although the benefit of intensively lowering blood glucose is unclear, newer antidiabetic drugs (glucagon‐like peptide‐1 receptor agonists and sodium‐glucose cotransporter‐2 inhibitors) have shown cardiovascular benefits in addition to their antihyperglycemic effect. Yet, studies suggest that recent use of evidence‐based therapy and management of cardiovascular risk among individuals with type 2 diabetes (T2D) and cardiovascular disease (CVD) remains largely suboptimal. The following narrative review first identifies barriers to translating research evidence to clinical practice at the levels of provider, health system, patient, and cost. Then it synthesizes previous implementation strategies that addressed multifaceted barriers and attempted to improve care for patients with T2D and CVD. In conclusion, team‐based care coordination, reminding systems in combination to pharmacist consultation and patient education, provider education compatible with clinical workflow, and coupled incentives between providers and patients appeared to be effective in reducing cardiovascular risks for patients with T2D and CVD, though the scalability and long‐term clinical effect of these strategies as well as the possibility of interventions involving payers and health systems remain uncertain.

## 1 INTRODUCTION

Type 2 diabetes (T2D) and cardiovascular disease (CVD) are the leading causes of morbidity and mortality in the United States.^1^ To varying extents, therapy that target major cardiovascular risk factors (dyslipidemia, hypertension, smoking, obesity, and hyperglycemia) have demonstrated benefits in improving cardiovascular outcomes over the past two decades. Cholesterol lowering, hypertension management, smoking cessation programs, and bariatric surgery have shown significant benefits in reducing cardiovascular adverse events among patients with T2D. Although conventional antihyperglycemic therapy have failed to improve long‐term macrovascular outcomes, two new classes of antidiabetic medications, glucagon‐like peptide‐1 receptor agonists (GLP1ra) and sodium‐glucose cotransporter‐2 inhibitors (SGLT2i), appear to exert macrovascular benefit among patients with T2D independent of their glycemic effect.^2^, ^3^

However, despite the substantial and emerging evidence of secondary preventive therapy, comprehensive cardiovascular risk reduction in patients with T2D and CVD remains suboptimal. Table 1 presents the percentages of US adults with T2D and CVD in the gaps of preventative care for five individual cardiovascular risk factors based on the National Health and Nutrition Examination Survey.^4^, ^5^ In particular, between 1999 and 2010, 78.7% of the adults with T2D and CVD did not achieve one or more guideline‐recommended goals for hemoglobin A1C (A1C), blood pressure, and low‐density lipoprotein cholesterol (LDL‐C); when obesity was included as a poorly managed risk factor, 90.6% of the patients were in the gaps of preventative care.^5^ Even in the COURAGE trial that included intensive medical therapy in both intervention arms to reduce cardiovascular risk, 56.9% of enrolled patients had more than four risk factors not at goal 1 year after randomization.^6^ Table 2 presents the percentages of patients in the COURAGE trial with diabetes and stable coronary disease not at goal for seven individual cardiovascular risk factors between randomization and 1 year after. Globally, the achievement of secondary prevention measures among patients with T2D and CVD varies by region. Data from the TECOS trial between 2008 and 2012 found that those in Eastern Europe and Latin America were more likely to have suboptimal LDL‐C levels (≥70 mg/dL) than those in North America.^7^ Across 38 countries, 42.1% of patients with diabetes and CVD had poorly controlled blood pressure (≥140 mm Hg systolic, ≥90 mm Hg diastolic). Even though current secondary cardiovascular prevention is suboptimal, previous studies have demonstrated the possibility and benefits of intensively and simultaneously managing multiple cardiovascular risk factors.^8^, ^9^

**Table 1:** Percentages of patients with T2D and CVD who have suboptimal management of five individual cardiovascular risk factors

	Time frames by years					
Suboptimal cardiovascular risk factors	1999‐2000	2001‐2002	2003‐2004	2005‐2006	2007‐2008	2009‐2010
LDL‐C ≥ 100 mg/dL, %	64.5	39.0	60.2	46.6	32.8	29.9
BP ≥ 130/80 mm Hg, %	73.9	58.7	68.8	56.6	54.8	50.8
BMI ≥ 25 kg/m^2^, %	89.7	87	83.2	91.4	81.7	91.2
Smoking, % (including those with and without CVD)	25	29	31	17	27	‐
HbA_1C_ ≥ 7%, %	25	29	31	17	27	25

*Note*: Adapted from Clair et al^4^ and Wong et al.^5^

Abbreviations: BP, blood pressure; BMI, body mass index; CVD, cardiovascular disease; HbA_1C_, hemoglobin A1c; LDL‐C, low density lipoprotein cholesterol; T2D, type 2 diabetes.

**Table 2:** Percentages of patients with diabetes and stable coronary disease who had suboptimal management for seven individual cardiovascular risk factors at the time of randomization and 1 year after

Cardiovascular risk factors	At the COURAGE trial randomization (n = 690)	1 year after (n = 592)
LDL‐C ≥ 85 mg/dL, %	41	41
SBP ≥ 130 mm Hg, %	57	46
BMI ≥ 25 kg/m^2^, %	90	90
Smoking, %	17	15
HbA_1C_ ≥ 7%, %	55	50
Physical activity < 150 min/week	81	61
Not adherent to AHA step 2 diet	41	18

Adapted from Mancini et al.^6^

Abbreviations: AHA, American Heart Association; BMI, body mass index; CVD, cardiovascular disease; HbA_1C_, hemoglobin A1c; LDL‐C, low density lipoprotein cholesterol; SBP, systolic blood pressure.

To provide insight on bridging the care gap, this narrative review outlines barriers and existing implementation strategies at the provider, health system, and patient levels, as well as issues related to cost in the context of T2D and CVD management.

## 2 BARRIERS TO EFFICACIOUS T2D AND CVD MANAGEMENT

### 2.1 Provider and system level barriers

#### 2.1.1 Insufficient provider education and clinical inertia

Although many stakeholders are involved in the process of translating research to practice, providers act as a vital force to mobilize the translation and prescribe evidence‐based therapy. Multiple studies have reported provider‐level barriers to achieving targets of cardiovascular risk factors such as inadequate awareness of guidelines and clinical inertia. According to a survey responded by 156 cardiologists and 149 cardiovascular teams who treat patients with dyslipidemia in 2017, 29% of cardiologists and 31% of cardiovascular team members lacked an understanding of guideline‐supporting evidence.^10^ Clinical inertia, which is the hesitancy of providers to initiate or intensify therapies despite evidence to do so, is another barrier associated with poor cardiovascular risk factor management. In a prospective cohort study of 1169 diabetic patients with elevated triage BP (>140/90 mm Hg), 51% of them did not have any treatment changed at their primary care visits.^11^

#### 2.1.2 Care coordination burden and poor community integration

Patients with T2D and CVD often require comanagement of several providers, including a primary care provider (PCP), cardiologist, and sometimes an endocrinologist. Yet, care coordination between these specialists can be constrained due to documentation burden on the electronic health record (EHR).^12^, ^13^ One study using the EHR logs of 471 primary care physicians found that physicians on average spent 3.2 hours per day on EHR documentation, of which less than 6% was on care coordination or making referrals.^12^ Even when patient care was coordinated across providers, poor communication among physicians could impose barriers to medical care. A survey of PCPs and specialists found that close to 50% of respondents reported a problem with the timeliness of referral information and that 30% were not satisfied with the referral content they received.^14^ Further, unmet social needs and inadequate community resources have been increasingly suggested as system‐level barriers to managing CVD and reducing healthcare disparities.^15^, ^16^ Therefore, developing effective education and care coordination strategies for clinicians who have limited office time is critical.

### 2.2 Patient level barriers

#### 2.2.1 Medication nonadherence

Nonadherence to medications is a common barrier to desired clinical outcomes at the patient level.^17^ Proportion of days covered (PDC) and medication possession ratio (MPR) are two valid and widely used measurements to evaluate medication adherence.^18^ A meta‐analysis of eight observational studies showed a 37.8% rate of poor adherence (PDC < 80%) to antihyperglycemic and cardiovascular drug therapy among adults with T2D.^19^ Similarly, a meta‐analysis of 19 cardiovascular prevention studies reported that 34% of patients with prior CVD adhered poorly (PDC < 75%) to five classes of guideline‐recommended medications.^20^ Among adults with a prior history of CVD, the overall adherence rate to evidence‐based medications was shown to improve modestly over time, though the adherence to different drug classes was highly heterogeneous, ranging between 40% and 80%.^21^, ^22^ The percentage of patients with prior myocardial infarction (MI) who fully adhered to statin, beta‐blocker, and angiotensin‐converting enzyme inhibitors or angiotensin receptor blockers (ACEI/ARB) increased from 29.1% in 1995 to 46.4% in 2003. In contrast, limited data exist regarding the adherence to GLP1ra and SGLT2i.

The reasons for nonadherence are multifaceted. One survey of over 24 000 adults with chronic illness found that up to 70% of patients reported at least one of three unintentional adherence behaviors: forgetting to take medication, forgetting to refill prescriptions, or not taking medication at the correct times.^23^ Further, patients with diabetes and cardiovascular risk factors were more likely to report unintentional nonadherence compared to those with only cardiovascular risk factors alone (odds ratio [OR] = 1.38; 95% CI, 1.25‐1.52). Despite heterogeneity in instruments, cross‐sectional studies found that patient perceptions such as concerns about medications, inconvenience, and emotional stress were associated with nonadherence among patients with diabetes or CVD.^24^, ^25^

#### 2.2.2 Patient‐physician mistrust

Distrust towards providers may hinder patients' desire to share their perspectives with providers, thus contributing to subsequent nonadherence. A survey of 80 females found that patient compliance differed significantly by different levels of trust in their PCPs (*P* = .015).^26^ Such an association might be modified by race/ethnicity: another cross‐sectional study of 723 hypertensive patients reported that white patients in race‐concordant provider relationships were more likely to be adherent compared with African American patients in race‐discordant provider relationships (OR = 1.27; 95% CI, 1.01‐1.61).^27^ In addition, prescription costs can considerably affect a patient's ability to self‐manage T2D and CVD.

### 2.3 Cost‐related issues

Out‐of‐pocket cost burden is one major barrier to use of evidence‐based medications. Multiple studies have highlighted the association between increased prescription costs and reduced use of evidence‐based medications including statins and beta‐blockers among patients with T2D and/or a history of CVD.^28^, ^29^, ^30^ Although GLP1ra and SGLT2i have cardiovascular benefits in addition to glycemic improvement, high prescription costs may significantly impede their use. For example, the monthly cost based on invoices from retail pharmacies ranged between $634 and $835 for GLP1ra injection pens (exenatide, dulaglutide, semaglutide, and liraglutide), and was approximately $450 for a 30‐day supply of SGLT2i tablets (dapagliflozin, canagliflozin, and empagliflozin).^31^

Another cost‐related barrier is the lengthy process of prior authorization, which is a utilization management strategy created by health payers and pharmacy benefit managers to contain high drug costs. American Medical Association conducted a survey in 2018 on how prior authorization affects clinical care and found that 86% of corresponding physicians felt high or extremely high burden with prior authorization and that 91% of them reported a negative impact on patient clinical outcomes due to prior authorization.^32^ High prescription costs can divert individuals from taking evidence‐based medications and the convoluted prior authorization process may be a possible reason of low‐use in GLP1ra and SGLT2i among patients with T2D and CVD.

## 3 IMPLEMENTATION STRATEGIES TO IMPROVE PREVENTIVE CARE IN PATIENTS WITH T2D AND CVD

### 3.1 Provider and system level

#### 3.1.1 Care coordination

Although many studies have attempted to improve T2D and CVD management through care coordination, its clinical effectiveness remains inconsistent. A summary analysis of 15 trials in patients predominantly with CVD and T2D reported that a nurse often played a key role in coordinating care by communicating with between PCPs and patients as well as reinforcing patients to improve adherence to pharmaceutical and lifestyle therapy. Fourteen of these trials testing care coordination strategies showed no significant reduction in hospitalizations, and all trials demonstrated no adherence improvement.^33^ However, in another randomized study of 214 patients with diabetes, coronary heart disease, or both, a goal‐driven care coordination plan where nurses closely monitored patients' progress, adjusted medications accordingly, and provided motivational coaching significantly led to a between‐group reduction in A1C by 0.58%, LDL‐C by 6.9 mg/dL, and systolic blood pressure by 5.1 mm Hg compared to controls (*P* < .001).^34^ Other evidence suggests that nurses or outreach coordinators can improve cardiovascular risk factor management by further reducing clinical inertia and healthcare costs.^35^ Given the complexity of care for patients with T2D and known CVD, care coordination between PCPs and specialists may also be effective, although limited data are available on how it should be designed and whether it improves clinical biomarkers.

#### 3.1.2 Provider education and clinical decision support

With the evolving evidence of new antihyperglycemic therapy and increasing burden for clinicians to translate a plethora of research literature into practice, clinical inertia and lack of familiarity on guideline‐based care can impede optimal T2D and CVD management. However, it is challenging to facilitate provider‐level education because of the heterogeneity of knowledge level, limited clinical time, and concerns about irritating providers when implementing such interventions.^33^ Therefore, clinical decision support systems have been suggested to play a role in recommending evidence‐based care while assisting busy clinicians. Yet, the benefit of decision support systems on clinical outcomes related to CVD and diabetes is not clear.^36^ One cluster‐randomized study evaluated the effect of an EHR‐based diabetes clinical decision support system on the control of cardiovascular risk factors vs usual care among 2556 patients with T2D across 11 primary care clinics.^37^ The clinical support system provided evidence‐based diabetes treatment options at the patient visit and reminded the physicians about medication changes, overdue lab tests, follow‐up intervals, and patients not at goal. After 6 months, the intervention clinics had a significant reduction in A1C (−0.26%; *P* = .01) and an increased proportion of patients whose systolic blood pressure was below 130 mm Hg (5.1%; *P* = .03) compared with the control clinics. No significant differences were found in the proportions of patients whose A1C, diastolic blood pressure, or LDL‐C reached optimal targets. Given clinicians' limited time and the breadth of T2D and CVD management, future provider‐level interventions need to be appropriately tailored to specific specialists and should not disrupt clinical workflow.

#### 3.1.3 Social integration

Social interventions such as guidance on community resources and provision of basic needs can also improve cardiovascular risk factor management. For example, the Health Leads program screened primary care patients for unmet basic needs such as food, medication, housing, and transportation; if screened positive, those patients (26% of whom had diabetes) would be connected to a patient advocate who helped patients to navigate community resources. Between pre‐and‐post intervention, and comparing to those screened negative, those screened positive had a significant reduction in systolic blood pressure (−1.6 mm Hg; 95% CI, −2.5 to −0.6 mm Hg) and LDL‐C (−3.9 mg/dL; 95% CI, −7.2 to −0.6 mg/dL), but not in A1C.^38^ The effect of such social interventions on secondary CVD has not been studied.

#### 3.1.4 Framework‐based interventions

Since barriers to optimal T2D and CVD management are multifaceted, several interventions have adopted a theoretical framework to improve health for patients with chronic diseases. One well‐established framework is chronic care model (CCM),^39^ a 6‐component model to improve chronic disease management. However, the effect of each independent component embedded in the CCM (community, health system, self‐management support, delivery system design, decision support, and clinical information systems) has not shown definite benefits on cardiovascular risk reduction.^40^, ^41^ A systematic review found that among 25 CCM‐based studies, the proportion of patients who reached desired cardiovascular risk factor targets ranged from 1.8% to 28% for A1C, 3.8% to 45% for blood pressure reduction, and 3.2% to 58% for optimal lipid control.^42^

Clinical decision support and framework‐based interventions seem to address provider and system‐level barriers to managing patients with T2D and CVD. However, their effect is unclear and future studies should focus on investigating clinical endpoints and improving workflow integration and sustainability. In contrast, care coordination programs where the nurse coordinator was able to discuss specific goals with patients, adjust medications, and closely monitor patients' progress showed success in T2D and CVD management. Social interventions can also improve disease management, but their feasibility and scalability may be a concern, particularly for patients with T2D and CVD who require more intensive healthcare resources than low cardiovascular risk patients.

### 3.2 Patient level

#### 3.2.1 Reminders and mobile technologies

Reminding systems have been implemented to target forgetfulness and address nonadherence to cardiometabolic medications among patients with T2D or CVD. However, the effect of reminding systems has thus far been minimal. The REMIND trial investigated the effect of three low‐cost reminder devices (pill bottle strip with toggles, digital timer cap, or standard pillbox) on improving medication adherence among 18 to 64‐year‐old adults who were taking one to three medications, yet with suboptimal adherence (MPR of 30%‐80%). Optimal adherence (MPR ≥ 80%) did not differ between any groups after 12 months, including those who were taking medications for cardiovascular or non‐depressive conditions.^43^ The IMAGE‐CHD trial examined the effect of two low‐literacy reminding strategies, an illustrated schedule and a postcard refill reminder, via a 2 × 2 factorial design in patients with prior coronary heart disease.^44^ There was no significant difference in medication adherence across all intervention arms compared with controls after 12 months. Finally, the HeartStrong trial randomized 1509 patients following an acute MI to an intervention that used an electronic pill bottle, lottery incentives, social support, and engagement counseling, vs usual care.^45^ There was no significant difference between the intervention and usual care in clinical outcomes or medication adherence after 1 year.

#### 3.2.2 Patient education and pharmacy‐based interventions

In contrast, pharmacy consultation combined with patient education has demonstrated benefits in improving adherence to cardiovascular medications and cardiovascular risk factor management, including diabetes care. The FAME trial was the first study to test the effect of a pharmacy‐based program that combined patient education, medication management, and regular follow‐ups with pharmacists on medication adherence and clinical outcomes among elderly patients.^46^ After a 6‐month run‐in period, the proportion of patients with good adherence (taking greater or equal to 80% of pills prescribed) in the entire study sample increased from 5.0% to 98.7% (*P* < .001). Following randomization and another 6 months of follow‐up, 97.4% of pills in the pharmacy‐based intervention arm were taken as instructed, whereas the percentage declined to 69.1% in the usual care arm (*P* < .001). Sustained adherence was associated with significant reductions in systolic blood pressure only in the intervention arm (−6.9 mm Hg; *P* = .001), but not in the usual care arm (−1.0 mm Hg; *P* = .69). Another pharmacist‐led intervention incorporated patient education, care coordination with PCPs, and reminding messages in discharged patients with acute coronary syndrome (45% of whom had diabetes).^47^ Compared with usual care, the intervention led to 15% more patients to adhere (PDC ≥ 80%) to four classes of cardiovascular medications (beta‐blockers, statins, antiplatelet agents, and ACEI/ARB, *P* = .003). However, blood pressure, LDL‐C, and costs did not differ significantly between the intervention and control arms after 12 months.

#### 3.2.3 Behavioral economics

Behavioral economics was also applied to couple incentives between providers and patients in order to improve lipid management in high cardiovascular risk population. One multicenter study randomized 340 primary care physicians and their 1503 patients (34% had preexisting coronary artery disease) to a physician incentive arm (quarterly payments based on monthly reports on patients' adherence and LDL‐C levels), patient incentive arm (a daily lottery), shared physician‐patient incentive arm (half of the benefits received in other two arms), or control.^48^ After 12 months, only the shared physician‐patient incentives group achieved a significant reduction in LDL‐C (8.5 mg/dL; 95% CI, 3.8‐13.3) compared with control. However, no other cardiovascular risk factors and clinical outcomes were evaluated in the study.

These patient‐level intervention studies suggest that reminders plainly addressing forgetfulness are unlikely to improve medication adherence. However, support from health professionals such as pharmacists, mediated through patient education, can improve medication adherence and some clinical outcomes in patients with T2D and CVD. Incentivized patient‐provider co‐management and smooth transitions between refills have also shown clinical benefits, yet whether these interventions are scalable and feasible in different health systems is unknown.

### 3.3 Cost‐related interventions

Interventions that have tested the effect of financial incentives on the management of patients with T2D and/or CVD reported mixed results. The Myocardial Infarction Free Rx Event and Economic Evaluation (MI FREEE) trial collaborated with an insurance sponsor to test the elimination of out‐of‐pocket costs for evidence‐based therapy among MI patients on adherence, clinical outcomes, and cost spending, compared with usual prescription coverage. In the full‐coverage group, the rate of adherence to ACEI/ARBs, beta‐blockers, and statins increased by 5.4% (95% CI, 3.6‐7.2, *P* < .001), compared with the usual‐coverage group.^49^ Although there was no significant reduction in the primary outcome (the rate of a fatal or nonfatal vascular event or revascularization) between groups, the full‐coverage group had a significant reduction in the rate of stroke (HR = 0.69; 95% CI, 0.50‐0.96). In terms of health spending, the mean total spending was $66 008 in the full‐coverage group and $71 778 in the usual‐coverage group (relative spending, 0.89; 95% CI, 0.50‐1.56). Another study examined the effect of a value‐based insurance policy on medication adherence among patient with diabetes or vascular disease. The policy of changing statin copayment from $24.18 to $0.60 was associated with an immediate 3.1% increase in monthly statin adherence and such increase sustained for the subsequent 10 months. In addition, when the monthly clopidogrel copayment reduced from $17.22 to $8.86, the policy change was associated with an immediate 4.2% increase in monthly adherence compared to control and the difference also sustained.^50^

Lowering prescription copayment has also been tested in patients after MI hospitalization who were recommended to use antiplatelet therapy for 1 year. The ARTEMIS trial randomized 11 001 patients with MI across 301 US hospitals to either the intervention group where copayment vouchers were provided to eliminate the prescription costs of clopidogrel or ticagrelor for 1 year, or the usual care group without vouchers.^51^ Medication persistence, defined as patient‐reported use of P2Y12 inhibitors without a gap of 30 days or longer, was significantly higher in the intervention group compared to control after adjusting for baseline characteristics (2.3%; 95% CI, 0.4‐4.1). However, there was no significant difference in the 3‐point composite MACE (death, MI, stroke) between the two groups.

**Figure 1:**
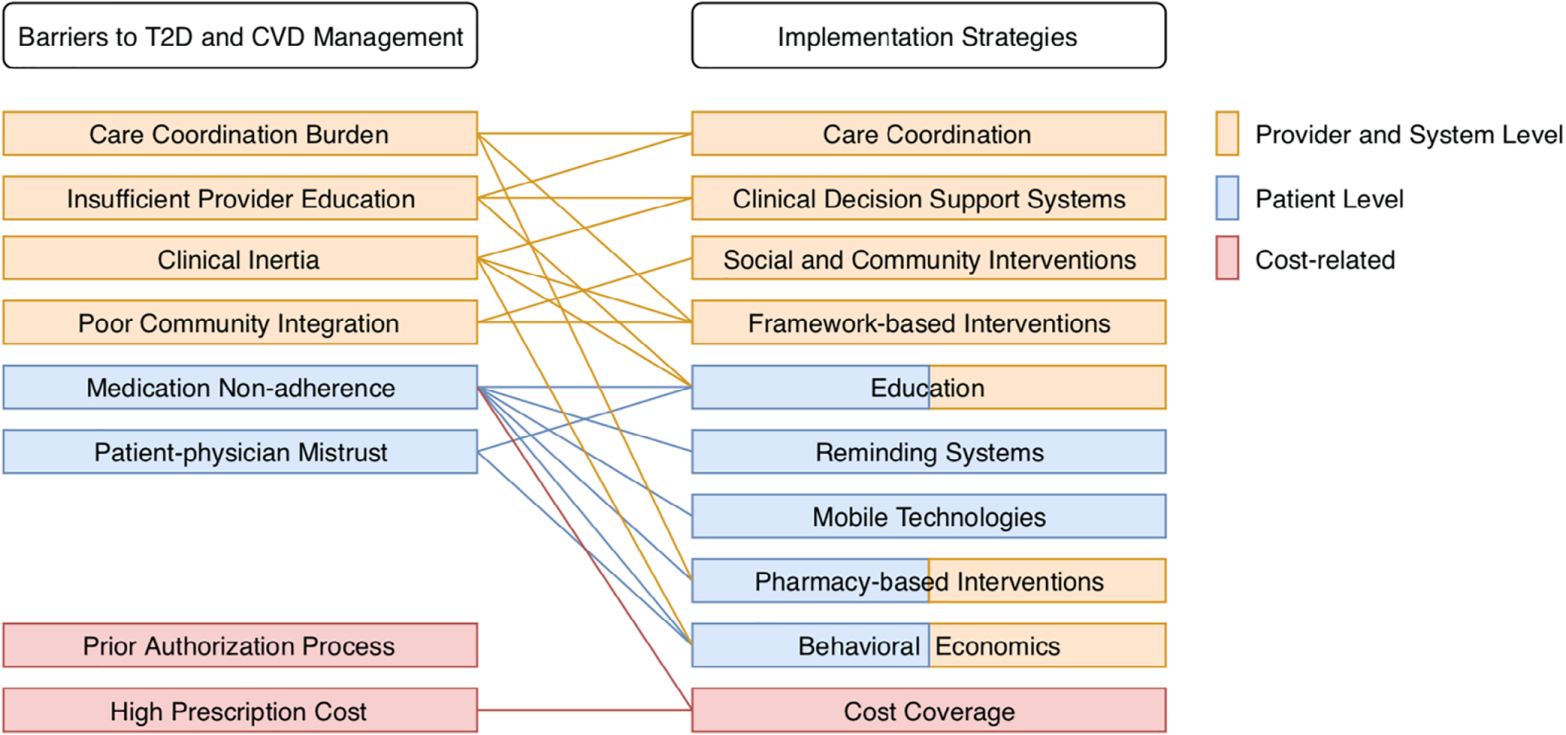
Framework of barriers to optimal management of T2D and CVD and corresponding implementation strategies. Provider and system level (orange), patient level (blue), and cost‐related (red). Abbreviations: CVD, cardiovascular disease; T2D, type 2 diabetes

Studies using novel approaches to remove cost‐related barriers have demonstrated potentials of improving patient adherence to guideline‐recommended therapy. The results also suggested benefits in clinical outcomes such as stroke incidence, yet the magnitude of improvement may not be clinically meaningful. Optimal T2D and CVD management is unlikely to be achieved unless interventions address cost barriers along with provider, health system, or patient‐level barriers. Figure 1 provides an overall visual summary of the barriers and corresponding implementation strategies to optimal management of T2D and CVD.

### 3.4 Next steps

Although observational studies and clinical trials have confirmed the efficaciousness of interventions that target multiple cardiovascular risk factors among patients with T2D and CVD, data from national surveys and multinational studies suggested that recent secondary prevention in this high‐risk population is largely inadequate, with some study population having as low as 2% achieving all goals for major cardiovascular risk factors.

Barriers to translating those study results to actual practice continue to exist on many levels. To date, various implementation interventions pertinent to T2D and CVD management were conducted to explore possible pathways. Specifically, nurse‐facilitated care coordination may help providers take a more active role in prescribing evidence‐based therapy to patients. Effective communication among providers, familiarity with new potent therapy, and interventions that address clinical inertia may improve care quality on the provider level. In addition, a comprehensive “team approach” consisting of PCPs, endocrinologists, cardiologists, pharmacists, nurses, dietitians, and other specialists was recommended by the 2018 ACC Expert Consensus Decision Pathway to optimally manage patients with T2D and CVD.^52^ On the patient side, adherence‐targeting interventions may be most effective when reminding systems are coupled with pharmacist consultation and patient education. Interactions between providers and patients appear to be also critical in improving patient adherence and cardiovascular risk factors.

Lastly, few implementation studies focused on the evaluation of multiple surrogate biomarkers, long‐term clinical endpoints, or cost‐effectiveness metrics. To drive clinical changes and downstream benefit on the population level, incentives for both providers and patients as well as collective efforts from payers and health systems may be required so that payment structure will be redesigned for preventative care in patients with T2D and CVD. The scalability and sustainability of many implementation strategies are worth being further assessed.

## CONFLICT OF INTEREST

The authors declare no potential conflict of interests.

## References

[1] Centers for Disease Control and Prevention . National Diabetes Statistics Report, 2017; 2017.

[2] Bethel MA , Patel RA , Merrill P , et al. Cardiovascular outcomes with glucagon‐like peptide‐1 receptor agonists in patients with type 2 diabetes: a meta‐analysis. Lancet Diabetes Endocrinol. 2018;6(2):105‐113. 29221659 10.1016/S2213-8587(17)30412-6

[3] Zelniker TA , Wiviott SD , Raz I , et al. SGLT2 inhibitors for primary and secondary prevention of cardiovascular and renal outcomes in type 2 diabetes: a systematic review and meta‐analysis of cardiovascular outcome trials. Lancet. 2019;393(10166):31‐39. 30424892 10.1016/S0140-6736(18)32590-X

[4] Clair C , Meigs JB , Rigotti NA . Smoking behavior among US adults with diabetes or impaired fasting glucose. Am J Med. 2013;126(6):541 e515‐541 e548. 10.1016/j.amjmed.2012.11.029 PMC4151048 23597801

[5] Wong ND , Patao C , Wong K , Malik S , Franklin SS , Iloeje U . Trends in control of cardiovascular risk factors among US adults with type 2 diabetes from 1999 to 2010: comparison by prevalent cardiovascular disease status. Diab Vasc Dis Res. 2013;10(6):505‐513. 23975724 10.1177/1479164113496828 PMC4227398

[6] Mancini GBJ , Maron DJ , Hartigan PM , et al. Lifestyle, glycosylated hemoglobin A1c, and survival among patients with stable ischemic heart disease and diabetes. J Am Coll Cardiol. 2019;73(16):2049‐2058. 31023428 10.1016/j.jacc.2018.11.067

[7] Pagidipati NJ , Navar AM , Pieper KS , et al. Secondary prevention of cardiovascular disease in patients with type 2 diabetes mellitus: international insights from the TECOS trial (trial evaluating cardiovascular outcomes with sitagliptin). Circulation. 2017;136(13):1193‐1203. 28626088 10.1161/CIRCULATIONAHA.117.027252 PMC5614823

[8] Gaede P , Lund‐Andersen H , Parving HH , Pedersen O . Effect of a multifactorial intervention on mortality in type 2 diabetes. N Engl J Med. 2008;358(6):580‐591. 18256393 10.1056/NEJMoa0706245

[9] Griffin SJ , Borch‐Johnsen K , Davies MJ , et al. Effect of early intensive multifactorial therapy on 5‐year cardiovascular outcomes in individuals with type 2 diabetes detected by screening (ADDITION‐Europe): a cluster‐randomised trial. Lancet. 2011;378(9786):156‐167. 21705063 10.1016/S0140-6736(11)60698-3 PMC3136726

[10] American College of Cardiology . Hypertriglyceridemia: insights on needs of cardiologists, CV team to reduce residual risk; 2018.

[11] Kerr EA , Zikmund‐Fisher BJ , Klamerus ML , Subramanian U , Hogan MM , Hofer TP . The role of clinical uncertainty in treatment decisions for diabetic patients with uncontrolled blood pressure. Ann Intern Med. 2008;148(10):717‐727. 18490685 10.7326/0003-4819-148-10-200805200-00004

[12] Tai‐Seale M , Olson CW , Li J , et al. Electronic health record logs indicate that physicians split time evenly between seeing patients and desktop medicine. Health Aff (Millwood). 2017;36(4):655‐662. 28373331 10.1377/hlthaff.2016.0811 PMC5546411

[13] Sinsky C , Colligan L , Li L , et al. Allocation of physician time in ambulatory practice: a time and motion study in 4 specialties. Ann Intern Med. 2016;165(11):753‐760. 27595430 10.7326/M16-0961

[14] Gandhi TK , Sittig DF , Franklin M , Sussman AJ , Fairchild DG , Bates DW . Communication breakdown in the outpatient referral process. J Gen Intern Med. 2000;15(9):626‐631. 11029676 10.1046/j.1525-1497.2000.91119.x PMC1495590

[15] Alley DE , Asomugha CN , Conway PH , Sanghavi DM . Accountable health communities – addressing social needs through Medicare and Medicaid. N Engl J Med. 2016;374(1):8‐11. 26731305 10.1056/NEJMp1512532

[16] Seligman HK , Laraia BA , Kushel MB . Food insecurity is associated with chronic disease among low‐income NHANES participants. J Nutr. 2010;140(2):304‐310. 20032485 10.3945/jn.109.112573 PMC2806885

[17] Osterberg L , Blaschke T . Adherence to medication. N Engl J Med. 2005;353(5):487‐497. 16079372 10.1056/NEJMra050100

[18] Lam WY , Fresco P . Medication adherence measures: an overview. Biomed Res Int. 2015;2015:217047. 26539470 10.1155/2015/217047 PMC4619779

[19] Khunti K , Seidu S , Kunutsor S , Davies M . Association between adherence to pharmacotherapy and outcomes in type 2 diabetes: a meta‐analysis. Diabetes Care. 2017;40(11):1588‐1596. 28801474 10.2337/dc16-1925

[20] Naderi SH , Bestwick JP , Wald DS . Adherence to drugs that prevent cardiovascular disease: meta‐analysis on 376,162 patients. Am J Med. 2012;125(9):882‐887 e881. 22748400 10.1016/j.amjmed.2011.12.013

[21] Choudhry NK , Setoguchi S , Levin R , Winkelmayer WC , Shrank WH . Trends in adherence to secondary prevention medications in elderly post‐myocardial infarction patients. Pharmacoepidemiol Drug Saf. 2008;17(12):1189‐1196. 18956426 10.1002/pds.1671 PMC2680489

[22] Newby LK , LaPointe NM , Chen AY , et al. Long‐term adherence to evidence‐based secondary prevention therapies in coronary artery disease. Circulation. 2006;113(2):203‐212. 16401776 10.1161/CIRCULATIONAHA.105.505636

[23] Gadkari AS , McHorney CA . Unintentional non‐adherence to chronic prescription medications: how unintentional is it really? BMC Health Serv Res. 2012;12:98. 22510235 10.1186/1472-6963-12-98 PMC3375198

[24] Allen LaPointe NM , Ou FS , Calvert SB , et al. Association between patient beliefs and medication adherence following hospitalization for acute coronary syndrome. Am Heart J. 2011;161(5):855‐863. 21570514 10.1016/j.ahj.2011.02.009

[25] Zullig LL , Shaw RJ , Crowley MJ , et al. Association between perceived life chaos and medication adherence in a postmyocardial infarction population. Circ Cardiovasc Qual Outcomes. 2013;6(6):619‐625. 24221839 10.1161/CIRCOUTCOMES.113.000435

[26] Abel WM , Efird JT . The association between trust in health care providers and medication adherence among Black women with hypertension. Front Public Health. 2013;1:66. 24350234 10.3389/fpubh.2013.00066 PMC3860006

[27] Schoenthaler A , Montague E , Baier Manwell L , Brown R , Schwartz MD , Linzer M . Patient‐physician racial/ethnic concordance and blood pressure control: the role of trust and medication adherence. Ethn Health. 2014;19(5):565‐578. 24266617 10.1080/13557858.2013.857764 PMC4031314

[28] Federman AD , Adams AS , Ross‐Degnan D , Soumerai SB , Ayanian JZ . Supplemental insurance and use of effective cardiovascular drugs among elderly Medicare beneficiaries with coronary heart disease. JAMA. 2001;286(14):1732‐1739. 11594898 10.1001/jama.286.14.1732

[29] Salas M , Hughes D , Zuluaga A , Vardeva K , Lebmeier M . Costs of medication nonadherence in patients with diabetes mellitus: a systematic review and critical analysis of the literature. Value Health. 2009;12(6):915‐922. 19402848 10.1111/j.1524-4733.2009.00539.x

[30] Goldman DP , Joyce GF , Escarce JJ , et al. Pharmacy benefits and the use of drugs by the chronically ill. JAMA. 2004;291(19):2344‐2350. 15150206 10.1001/jama.291.19.2344

[31] American Diabetes Association . 9. Pharmacologic approaches to glycemic treatment: standards of medical care in diabetes – 2019. Diabetes Care. 2019;42(suppl 1):S90‐S102. 30559235 10.2337/dc19-S009

[32] American Medical Association . 2018 AMA prior authorization (PA) physician survey; 2019.

[33] Peikes D , Chen A , Schore J , Brown R . Effects of care coordination on hospitalization, quality of care, and health care expenditures among Medicare beneficiaries: 15 randomized trials. JAMA. 2009;301(6):603‐618. 19211468 10.1001/jama.2009.126

[34] Katon WJ , Lin EH , Von Korff M , et al. Collaborative care for patients with depression and chronic illnesses. N Engl J Med. 2010;363(27):2611‐2620. 21190455 10.1056/NEJMoa1003955 PMC3312811

[35] Huebschmann AG , Mizrahi T , Soenksen A , Beaty BL , Denberg TD . Reducing clinical inertia in hypertension treatment: a pragmatic randomized controlled trial. J Clin Hypertens (Greenwich). 2012;14(5):322‐329. 22533659 10.1111/j.1751-7176.2012.00607.x PMC3340617

[36] Anchala R , Pinto MP , Shroufi A , et al. The role of decision support system (DSS) in prevention of cardiovascular disease: a systematic review and meta‐analysis. PLoS One. 2012;7(10):e47064. 23071713 10.1371/journal.pone.0047064 PMC3468543

[37] O'Connor PJ , Sperl‐Hillen JM , Rush WA , et al. Impact of electronic health record clinical decision support on diabetes care: a randomized trial. Ann Fam Med. 2011;9(1):12‐21. 21242556 10.1370/afm.1196 PMC3022040

[38] Berkowitz SA , Hulberg AC , Standish S , Reznor G , Atlas SJ . Addressing unmet basic resource needs as part of chronic cardiometabolic disease management. JAMA Intern Med. 2017;177(2):244‐252. 27942709 10.1001/jamainternmed.2016.7691 PMC6020679

[39] Wagner EH , Austin BT , Davis C , Hindmarsh M , Schaefer J , Bonomi A . Improving chronic illness care: translating evidence into action. Health Aff (Millwood). 2001;20(6):64‐78. 11816692 10.1377/hlthaff.20.6.64

[40] Smith SA , Shah ND , Bryant SC , et al. Chronic care model and shared care in diabetes: randomized trial of an electronic decision support system. Mayo Clin Proc. 2008;83(7):747‐757. 18613991 10.4065/83.7.747

[41] Chen EH , Thom DH , Hessler DM , et al. Using the Teamlet model to improve chronic care in an academic primary care practice. J Gen Intern Med. 2010;25(suppl 4):S610‐S614. 20737236 10.1007/s11606-010-1390-1 PMC2940441

[42] Yeoh EK , Wong MCS , Wong ELY , et al. Benefits and limitations of implementing chronic care model (CCM) in primary care programs: a systematic review. Int J Cardiol. 2018;258:279‐288. 29544944 10.1016/j.ijcard.2017.11.057

[43] Choudhry NK , Krumme AA , Ercole PM , et al. Effect of reminder devices on medication adherence: the REMIND randomized clinical trial. JAMA Intern Med. 2017;177(5):624‐631. 28241271 10.1001/jamainternmed.2016.9627 PMC5470369

[44] Kripalani S , Schmotzer B , Jacobson TA . Improving medication adherence through graphically enhanced interventions in coronary heart disease (IMAGE‐CHD): a randomized controlled trial. J Gen Intern Med. 2012;27(12):1609‐1617. 22790614 10.1007/s11606-012-2136-z PMC3509298

[45] Volpp KG , Troxel AB , Mehta SJ , et al. Effect of electronic reminders, financial incentives, and social support on outcomes after myocardial infarction: the HeartStrong randomized clinical trial. JAMA Intern Med. 2017;177(8):1093‐1101. 28654972 10.1001/jamainternmed.2017.2449 PMC5710431

[46] Lee JK , Grace KA , Taylor AJ . Effect of a pharmacy care program on medication adherence and persistence, blood pressure, and low‐density lipoprotein cholesterol: a randomized controlled trial. JAMA. 2006;296(21):2563‐2571. 17101639 10.1001/jama.296.21.joc60162

[47] Ho PM , Lambert‐Kerzner A , Carey EP , et al. Multifaceted intervention to improve medication adherence and secondary prevention measures after acute coronary syndrome hospital discharge: a randomized clinical trial. JAMA Intern Med. 2014;174(2):186‐193. 24247275 10.1001/jamainternmed.2013.12944

[48] Asch DA , Troxel AB , Stewart WF , et al. Effect of financial incentives to physicians, patients, or both on lipid levels: a randomized clinical trial. JAMA. 2015;314(18):1926‐1935. 26547464 10.1001/jama.2015.14850 PMC5509443

[49] Choudhry NK , Avorn J , Glynn RJ , et al. Full coverage for preventive medications after myocardial infarction. N Engl J Med. 2011;365(22):2088‐2097. 22080794 10.1056/NEJMsa1107913

[50] Choudhry NK , Fischer MA , Avorn J , et al. At Pitney Bowes, value‐based insurance design cut copayments and increased drug adherence. Health Aff (Millwood). 2010;29(11):1995‐2001. 21041738 10.1377/hlthaff.2010.0336

[51] Wang TY , Kaltenbach LA , Cannon CP , et al. Effect of medication co‐payment vouchers on P2Y12 inhibitor use and major adverse cardiovascular events among patients with myocardial infarction: the ARTEMIS randomized clinical trial. JAMA. 2019;321(1):44‐55. 30620370 10.1001/jama.2018.19791 PMC6583585

[52] Das SR , Everett BM , Birtcher KK , et al. 2018 ACC expert consensus decision pathway on novel therapies for cardiovascular risk reduction in patients with type 2 diabetes and atherosclerotic cardiovascular disease: a report of the American College of Cardiology Task Force on expert consensus decision pathways. J Am Coll Cardiol. 2018;72(24):3200‐3223. 30497881 10.1016/j.jacc.2018.09.020 PMC7560953

